# Lung hyperaeration assessment by computed tomography: correction of reconstruction-induced bias

**DOI:** 10.1186/s12871-016-0232-z

**Published:** 2016-08-24

**Authors:** Lorenzo Ball, Claudia Brusasco, Francesco Corradi, Francesco Paparo, Alessandro Garlaschi, Peter Herrmann, Michael Quintel, Paolo Pelosi

**Affiliations:** 1 Department of Surgical Sciences and Integrated Diagnostics, IRCCS AOU San Martino-IST, University of Genoa, Genoa, Italy; 2 Anaesthesia and Intensive Care, E.O. Ospedali Galliera, Genova, Italy; 3 Radiology Department, E.O. Ospedali Galliera, Genova, Italy; 4 Dipartimento di Diagnostica per Immagini, IRCCS-Azienda Ospedaliera Universitaria-IST, Genova, Italy; 5 Department of Anaesthesiology, Emergency and Intensive Care Medicine, University of Göttingen Medical Center, Göttingen, Germany

## Abstract

**Background:**

Computed tomography (CT) reconstruction parameters, such as slice thickness and convolution kernel, significantly affect the quantification of hyperaerated parenchyma (V_HYPER_%). The aim of this study was to investigate the mathematical relation between V_HYPER_% calculated at different reconstruction settings, in mechanically ventilated and spontaneously breathing patients with different lung pathology.

**Methods:**

In this retrospective observational study, CT scans of patients of the intensive care unit and emergency department were collected from two CT scanners and analysed with different kernel-thickness combinations (reconstructions): 1.25 mm soft kernel, 5 mm soft kernel, 5 mm sharp kernel in the first scanner; 2.5 mm slice thickness with a smooth (B41s) and a sharp (B70s) kernel on the second scanner. A quantitative analysis was performed with Maluna® to assess lung aeration compartments as percent of total lung volume. CT variables calculated with different reconstructions were compared in pairs, and their mathematical relationship was analysed by using quadratic and power functions.

**Results:**

43 subjects were included in the present analysis. Image reconstruction parameters influenced all the quantitative CT-derived variables. The most relevant changes occurred in the hyperaerated and normally aerated volume compartments. The application of a power correction formula led to a significant reduction in the bias between V_HYPER_% estimations (*p* < 0.001 in all cases). The bias in V_HYPER_% assessment did not differ between lung pathology nor ventilation mode groups (*p* > 0.15 in all cases).

**Conclusions:**

Hyperaerated percent volume at different reconstruction settings can be described by a fixed mathematical relationship, independent of lung pathology, ventilation mode, and type of CT scanner.

## Background

Lung quantitative computed tomography (CT) is an effective method for the evaluation of lung aeration, especially in the mechanically ventilated patients, since the quantification of over and non-aerated lung might be useful for the optimization of the ventilatory strategy [1]. Tidal recruitment and hyperaeration have been proposed as the main mechanisms yielding Ventilator Induced Lung Injury [2, 3], and can also occur at low tidal volume ventilation [3]. Mechanical ventilation should be individually titrated in order to avoid lung over distension, maintaining an acceptable gas exchange [4, 5].

However, the potential future of CT assessment of hyperaeration in clinical and research applications is still hampered by technical issues; in particular: 1) the optimal attenuation threshold separating normally from over-inflated lung parenchyma in mechanically ventilated patients is matter of debate [6]; 2) the reconstruction parameters, such as slice thickness and convolution kernel, significantly affect CT assessment of overinflated areas in both chronic emphysema [7] and critically ill patients [8]. Recent studies found a relevant amount of hyperaerated lung parenchyma among healthy patients [9], as well as a dramatic effect of reconstruction parameters on the evaluation of severe emphysema [10]. Thus, a standardized and reproducible evaluation of hyperaeration by CT is warranted. A new method has been recently proposed to correct the discrepancy in hyperaeration assessment at different CT reconstruction parameters in spontaneously breathing patients with chronic emphysema [11].

The aim of the present study was to develop a technique to standardize hyperaeration assessment in spontaneously breathing and mechanically ventilated patients with various underlying lung conditions, using different reconstruction settings and two different CT scanners. We hypothesized that hyperaerated percent volume at different reconstruction settings could be described by a fixed mathematical relationship, independent of lung pathology and ventilation mode.

## Methods

This was a retrospective observational study, reported according to the Strengthening the Reporting of Observational Studies in Epidemiology (STROBE) statement [12].

### Population

Patients of the Intensive Care Unit and the Emergency Department of a single university hospital in Genova, Italy, that underwent chest CT scan for clinical reasons between September 1^st^ and November 31^st^ 2012 were retrospectively screened for inclusion analysing the scanners’ database. The inclusion criteria were the following: 1) patient age ≥ 18 years; 2) standard helical thorax acquisition protocols used; 3) no contrast medium used and 4) no relevant motion artefacts.

### CT image acquisition and analysis

CT scans were collected from two different scanners, each patient underwent a single CT scan with either scanner A or B. Scanner A was a LightSpeed 16 (GE Medical Systems, Milwaukee, US), located in the emergency department, set with 120 kVp, field of view according to clinical needs (range 360–500), pitch factor 1.75, collimation 16 × 0.625 mm. The images of scanner A were reconstructed in axial DICOM series with three different kernel-thickness combinations (reconstructions): 1.25 mm soft kernel, 5 mm soft kernel, 5 mm sharp kernel. Smooth and sharp filters were the GE proprietary *Lung* and *Body* filters, respectively. Scanner B was a Somatom Emotion 6 (Siemens, Munich, Germany), located in the radiology department, and settings were: 110 kVp, Pitch Factor 0.85, Slice Collimation 6 × 2.0 mm. The images of scanner B were reconstructed at 2.5 mm slice thickness with a smooth (B41s) and a sharp (B70s) kernel. Scans were executed in accordance to the routine practice of our Radiology Unit: during breath-hold at full inspiration in spontaneously breathing patients, or during uninterrupted ventilation in mechanically ventilated patients.

Image segmentation was performed with Maluna® software (University of Mannheim, Göttingen, Germany) by means of a semi-automatic method with further manual refinement. Big vessels and airways larger than 5 mm were excluded from segmentation. Scans were segmented on all slices of each reconstruction by three physicians (LB, CB, and FC) and then revised by a radiologist (AG).

For each reconstruction, quantitative analysis was performed to obtain Total Lung Volume (TLV), mean lung attenuation in Hounsfield Units (HU), Total Lung Weight and the following aeration compartments [13] as % of TLV: hyper-aerated volume (V_HYPER_%, -1000 to -901 HU), normally aerated volume (V_NORMAL_%, -900 to -501 HU), poorly aerated volume (V_POOR_%, -500 to -101 HU), non-aerated volume (V_NON_ %, -100 to +100 HU).

CT variables calculated with the different CT reconstruction parameters were compared in pairs. For each reconstruction pair, V_HYPER_% was calculated at the two reconstructions and the mathematical relationship between the two was analysed by using a quadratic and power function according to the corrected Akaike information criterion (AICc), as previously described [11]. Patients were divided in three pathology groups according to the main finding of the CT scan: no pathological findings in lung parenchyma of a spontaneously breathing or mechanical ventilated patient (Healthy), hyperaerated lung parenchyma and findings compatible with chronic obstructive pulmonary disease (COPD), evidence of lung injury or aeration loss (Injury). Patients with COPD exacerbation were included in the Injury group due to the relevant amount of non-aerated lung volume.

### Statistical analysis

Data are presented as mean ± standard deviation where not otherwise specified; absolute volumes, weights and mean HU were rounded to the nearest integer. Normality was assessed by D’Agostino–Pearson omnibus test and comparisons were made by repeated measures t-test and repeated measures ANOVA with Tukey post-hoc or Wilcoxon tests and Friedman test with Dunn post-hoc, accordingly. Independent samples were compared by t-test or Kruskal-Wallis test. Bias between measurements of V_HYPER_% with the two reconstructions was compared before and after the application of such correction by Bland-Altman method. Expecting a very strong correlation (R ≥ 0.8) between V_HYPER_ calculated at different reconstructions [11], we needed to enrol at least 9 subjects from each scanner to achieve 90 % power (1-β) to observe such correlation at an alpha level of 0.05. Statistical analysis was performed with SPSS version 21 (IBM Corp.), function fit and graphs with Prism 6 (GraphPad Software Inc., San Diego, CA). Statistical significance was considered for *p* < 0.05, all tests were two-tailed.

## Results

In the observed period, 178 chest CT scans were performed. Among them, 135 were excluded: 5 patients were minors, 28 scans used non-standard acquisition protocols, 89 required the use of contrast medium and 13 reported unacceptable motion artefacts. As a result, 43 scans were included in the final analysis: 33 from scanner A and 10 from scanner B. The resulting population is described in Table 1.

**Table 1:** Patient data

Patient Data			
Age, y (IQR)	62 (47–71)		
Sex (%)	27 Male (62.8), 16 Female (37.2)		
Lung CT indication (%)	Respiratory Failure	27	*(62.8)*
	Polytrauma	6	*(14.0)*
	Other	10	*(23.3)*
Mode of ventilation (%)	Mechanically Ventilated	15	*(34.9)*
	Spontaneously Breathing	28	*(65.1)*
Main lung CT finding (%)	Injury	23	*(53.4)*
	COPD	10	*(23.3)*
	Healthy	10	*(23.3)*

**Fig. 1:**
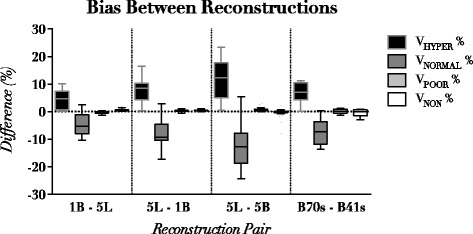
Differences between volume compartments assessment in different reconstruction pairs. GE LightSpeed: 1B (1.25 mm Body smooth kernel) 5B (5 mm Body smooth kernel) 5 L (5 mm Lung sharp kernel). Siemens Emotion: 2.5 mm sharp (B70s) and 2.5 mm smooth (B41s)

Image reconstruction parameters influenced all the quantitative CT-derived variables (see Table 2). The most relevant changes occurred in the V_HYPER_% and V_NORMAL_% volume compartments, while differences in Total Lung Volume, Lung Density, Lung Weight, V_POOR_% and V_NON_ % were limited in magnitude. As shown in Fig. 1, differences in V_HYPER_% were associated to opposite changes in V_NORMAL_% and the sum of the two compartments was not influenced by reconstruction parameters (*p* > 0.05 for all reconstruction pairs).

**Table 2:** Mean results of CT quantitative analysis at different slice thickness-kernel combinations for GE LightSpeed scanner and Siemens Emotion

GE LightSpeed									
Reconstruction		Total Volume (ml)	EI_950_%^a^	V_HYPER_ %^a^	V_NORMAL_ %^a^	V_POOR_ %^a^	V_NON_ %	Density (HU)^a^	Lung Weight (g)^a^
1.25 mm	*Body*	4021 ± 1494	1.0 ± 1.8	12.6 ± 14.3	72.7 ± 15.5	6.9 ± 5.0	7.0 ± 13.0	−721 ± 144	1013 ± 435
5.00 mm	*Body*	4037 ± 1500	0.3 ± 0.7	8.2 ± 11.8	77.3 ± 15.6	7.2 ± 5.3	6.6 ± 13.2^b^	−713 ± 146	1049 ± 464
5.00 mm	*Lung*	3943 ± 1451^b^	4.4 ± 4.3	20.3 ± 16.0	64.6 ± 13.9	7.6 ± 5.2	6.6 ± 11.7	−728 ± 139	961 ± 374
Siemens Somatom									
Reconstruction		Total Volume (ml)	EI_950_%^a^	V_HYPER_ %^a^	V_NORMAL_ %^a^	V_POOR_ %	V_NON_ %	Density (HU)^a^	Lung Weight (g)^a^
2.50 mm	*B41s*	3224 ± 1264	0.3 ± 0.4	5.0 ± 5.6	73.4 ± 14.8	14.3 ± 11.4	7.0 ± 7.0	−663 ± 136	960 ± 252
2.50 mm	*B70s*	3203 ± 1276	2.9 ± 2.3	12.0 ± 8.4	66.2 ± 11.6	14.7 ± 11.3	6.6 ± 6.1	−671 ± 130	933 ± 256

Repeated measures pairwise comparisons: ^a^all pairwise comparisons between reconstructions significant (*p* < 0.05), ^b^significantly different compared pairwise to both other reconstructions (*p* < 0.05)

**Fig. 2:**
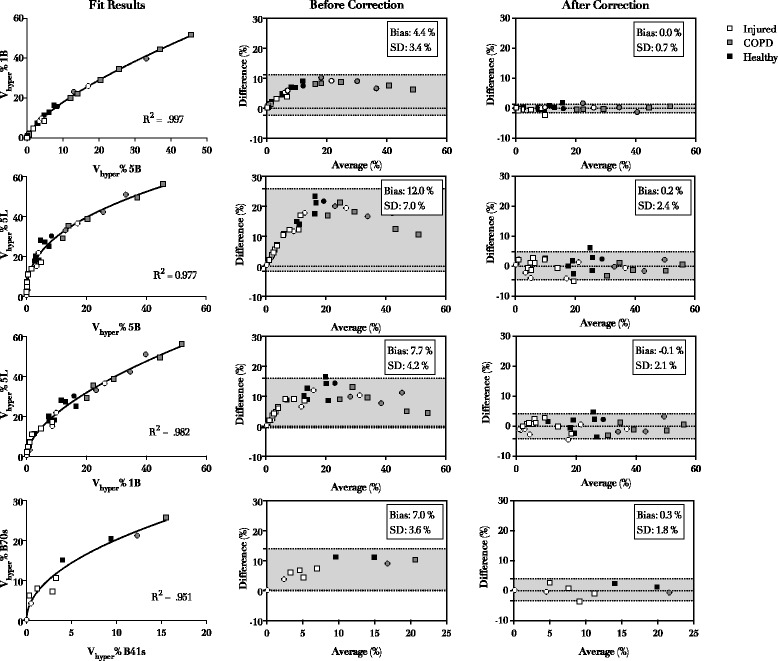
Fit results (*left panels*), agreement before (*middle panels*) and after (*right panels*) application of correction. Each panel row represents one reconstruction pair. GE LightSpeed 16: 1B (1.25 mm Body smooth kernel) 5B (5 mm Body smooth kernel) 5 L (5 mm Lung sharp kernel). Siemens Emotion 6: 2.5 mm sharp (B70s) and 2.5 mm smooth (B41s). Dot shapes indicates ventilation mode: spontaneously breathing (*squares*) and mechanically ventilated subjects (*circles*)

**Fig. 3:**
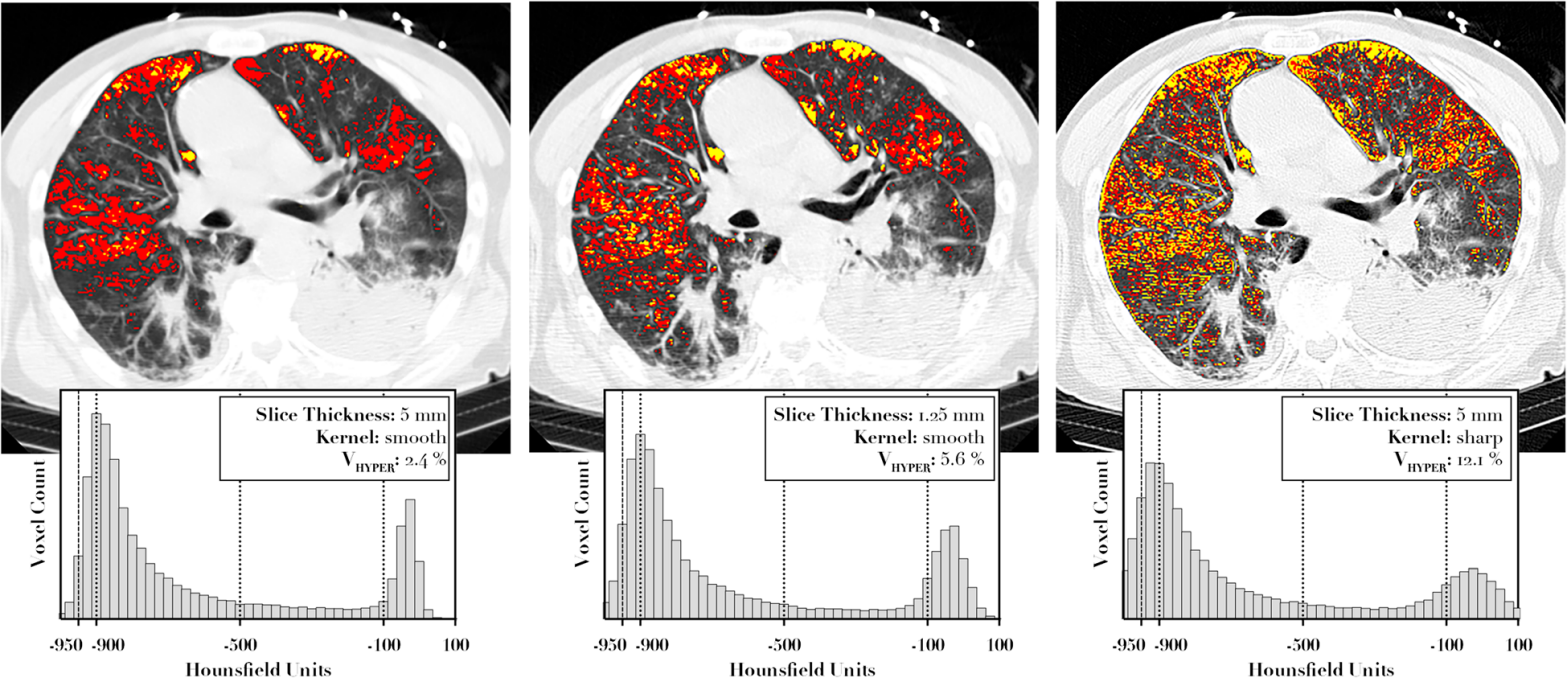
Effects of changes of slice thickness and kernel in distribution and amount of V_HYPER_% (*red mask*). Hyperaeration calculated with -950 HU as threshold is also shown (*yellow mask*)

For each reconstruction pair, the relationship between V_HYPER_% calculated in the two reconstructions was adequately described by both quadratic and power functions, and the preferred model according to the AICc was the power function (y = a · x^b^) in all cases, as shown in Table 3. The application of the correction formula led to a significant reduction in the bias between reconstructions in V_HYPER_% estimation (*p* < 0.001 in all cases), as shown in Fig. 2. Figure 3 illustrates the effect of reconstruction of a representative subject with injured lung, whose CT was acquired with scanner A and reconstructed with three different kernel-thickness combinations. The bias between reconstructions in V_HYPER_% assessment did not differ between lung pathology nor ventilation mode groups (see Table 4).

**Table 3:** Results of function fit. Parameters reported as mean ± standard error

	Reconstruction 1	Reconstruction 2	Best Model	a	b	R^2^
GE LightSpeed	5 mm body	1.25 mm body	*power function*	3.66 ± 0.12	0.69 ± 0.01	0.997
	5 mm body	5 mm lung	*power function*	11.60 ± 0.58	0.41 ± 0.02	0.977
	1. 25 mm body	5 mm lung	*power function*	5.64 ± 0.39	0.58 ± 0.02	0.982
Siemens Emotion	2.5 mm B70s	2.5 mm B41s	*power function*	6.58 ± 0.85	0.48 ± 0.06	0.951

**Table 4:** Bias between reconstructions in V_HYPER_% after correction, in the different lung pathology and ventilation mode groups

	Reconstruction 1	Reconstruction 2	Injured	COPD	Healthy	p
GE LightSpeed	5 mm body	1.25 mm body	0.2 ± 0.6	−0.1 ± 0.9	−0.3 ± 0.7	0.27
	5 mm body	5 mm lung	0.2 ± 2.4	0.4 ± 1.7	−1.6 ± 2.8	0.18
	1. 25 mm body	5 mm lung	0.3 ± 1.9	0.5 ± 2.0	−0.8 ± 2.7	0.39
Siemens Emotion	2.5 mm B70s	2.5 mm B41s	0.2 ± 2.1	−0.3 ± 1.4	−1.8 ± 0.8	0.45
	Reconstruction 1	Reconstruction 2	Spontaneously Breathing	Mechanically Ventilated		p
GE LightSpeed	5 mm body	1.25 mm body	0.1 ± 0.8	−0.1 ± 0.7		0.42
	5 mm body	5 mm lung	−0.4 ± 2.5	0.2 ± 2.3		0.54
	1. 25 mm body	5 mm lung	−0.3 ± 2.1	0.8 ± 2.0		0.15
Siemens Emotion	2.5 mm B70s	2.5 mm B41s	−0.5 ± 2.2	0.3 ± 0.4		0.38

## Discussion

The main findings of the present study are: 1) CT reconstruction mainly affected the estimation of V_HYPER_% and V_NORMAL_%; 2) the bias between reconstructions is mathematically determined and can be described by a power function; 3) such mathematical relationship can be used to correct the bias between two reconstructions, independent of lung pathology and ventilation mode.

In the last decade, several techniques have been introduced and investigated to assess at the bedside lung aeration, including lung ultrasound (LUS) [14, 15] and electrical impedance tomography (EIT) [16]. The latter is able to provide an estimation of hyper-aeration [17]. Nonetheless, CT remains the gold standard because of its strong correlation with physical density [15], and in the last decades it provided precious pathophysiological information in acute respiratory distress syndrome (ARDS) [18], therefore studies validating emerging monitoring techniques such as LUS and EIT often use CT as reference [19]. Thus, a standardization of hyperaeration measurement by CT is mandatory allowing the analysis and comparison of previously acquired datasets.

The present study shows that hyperaeration measured with a given slice thickness-kernel combination can be estimated using data from a different CT reconstruction. To our knowledge, this is the first study attempting to correct hyperaeration assessment bias in injured lung and during mechanical ventilation. A similar approach was proposed for spontaneously breathing chronic emphysema patients [11]. Using a comparable method, we found a similar performance on reconstruction bias correction in two different manufacturer scanners and in patients with different lung findings as well as type of ventilation. The discrepancy between quantitative CT analyses was primarily found in the hyperaerated and the normally aerated regions of the lung, as previously described [7, 8]. Thus, a single correction formula for V_HYPER_% can virtually completely eliminate bias between reconstructions, being applied directly to V_HYPER_%, and by subtraction to V_NORMAL_%. The hyperaeration assessment was initially studied by pulmonologists for staging chronic emphysema [20]. In this domain, though not unanimous, there is agreement that specific reconstructions, such as smooth B30s kernel wit 1 mm slice thickness or comparable, should be preferred to others due to their good correlation with quantitative histology [21, 22], using a threshold of -950 HU to define hyperaerated regions [11, 23]. In intensive care applications of lung CT analysis, quantification of hyperaeration is a functional and physiological rather than an anatomical assessment [6], thus a direct comparison with a morphological characteristic of the lung is not viable, and a threshold of -900 HU was usually employed [1, 4, 9, 13]. Due to the lack of a gold standard for comparison, safe amounts of hyperaeration at CT analysis are difficult to be determined [3, 4]. The results of the present work show that V_HYPER_ % calculated with different reconstructions can be compared once corrected by a mathematical formula. Other studies proposed to extrapolate quantitative CT analysis from a limited number of slices, potentially reducing dose exposure [24, 25]. We propose a mathematical allowing switching to thicker or thinner slices as needed without affecting V_HYPER_% measurement.

Applications of our method has relevance mainly in the research field, with potential indirect relevant clinical implications. Efforts should be made in order to obtain a standard CT assessment of hyper-aeration, as in recent experimental studies it was correlated with an increased neutrophilic inflammation in a model of ARDS [26]. CT scans performed for clinical reasons in critically ill patients often require the administration of contrast medium: this caused the exclusion of half of the screened patients in the present cohort. Due to the minimal perfusion of hyper-aerated lung regions, one could hypothesise that limited changes will occur in this compartment due to the presence of iodine; however, the validity of our method in CT scans acquired with contrast medium remains to be tested.

Our study has some limitations to be addressed. First, the retrospective design of the study led to limited possibility of controlling the correct execution of the chest scan, which was evaluated *a posteriori* by visual inspection of the images. Second, mechanically ventilated patients were not examined during a respiratory hold leading to artefacts and possibly inaccurate absolute estimation of V_HYPER_%. However, since in this study no comparison was performed between subjects but only on different CT scan reconstructions of the same patient, the mentioned limits should not affect the validity of the results. Third, reported correction formulas cannot be directly applied on other scanners, for which further calibration would be necessary to apply externally the findings of our study. Moreover, this technique, while able to reduce notably the discrepancy between V_HYPER_% estimation with two different reconstructions, is unable to clarify which specific reconstruction settings should be chosen: further studies are warranted to identify those that better correlate with histological findings, inflammation markers and, potentially, clinical outcomes.

## Conclusions

In quantitative lung CT, the relationship between hyperaerated percent volume at different slice thickness-convolution kernel settings can be described by a single function, independent of lung pathology, ventilation mode. This approach was feasible in the two different CT scanners used in the present study.

## Abbreviations

CT: Computed tomography
VILI: Ventilator-induced lung injury
ARDS: Acute respiratory distress syndrome
COPD: Chronic obstructive pulmonary disease
TLV: Total lung volume
HU: Hounsfield units
V_HYPER_%: Percent volume of hyper-aerated lung tissue
V_NORMAL_%: Percent volume of normally aerated lung tissue
V_POOR_ %: Percent volume of poorly aerated lung tissue
V_NON_%: Percent volume of non-aerated lung tissue
